# Bioluminescent imaging of *Arabidopsis thaliana* using an enhanced Nano-lantern luminescence reporter system

**DOI:** 10.1371/journal.pone.0227477

**Published:** 2020-01-03

**Authors:** Yuichi Furuhata, Ayako Sakai, Tomi Murakami, Akira Nagasaki, Yoshio Kato

**Affiliations:** Biomedical Research Institute, National Institute of Advanced Industrial Science and Technology (AIST), Tsukuba, Japan; Advanced Centre for Treatment Research and Education in Cancer, INDIA

## Abstract

Bioluminescent detection has become a powerful method that is used extensively in numerous areas in life science research. Given that fluorescence detection in plant cells is difficult owing to the autofluorescence of chlorophyll, the use of a luciferin–luciferase system should be effective in plant biology. However, the suitable optical window for a luminescence system in plants remains unexplored. In this study, we sought to determine the optical window and optimal luciferase reporter system for terrestrial plant analyses using *Arabidopsis thaliana* as a model organism. We compared six different luciferase systems and found the green enhanced Nano-lantern (GeNL)–furimazine combination to be the optimal luciferase reporter. Spectral measurements of GeNL–furimazine showed that its luminescence peak falls within the range of optical transparency for chlorophyll and, therefore, enables greater penetration through a layer of cultured *A*. *thaliana* cells. Moreover, *A*. *thaliana* plants expressing GeNL with furimazine emitted strong luminescence, which could be detected even with the naked eye. Thus, the GeNL–furimazine combination should facilitate biological analyses of genes and cellular functions in *A*. *thaliana* and all other terrestrial plants.

## Introduction

Luciferase has been used as a useful reporter gene for both in vitro and in vivo bioluminescent imaging to assess biological events in different organisms [1–5]. Various types of luciferase genes have been discovered or developed, including firefly, *Renilla*, and *Vargula* luciferases [1,3–6]. These systems can be classified according to the type of luciferin employed [1,3–6]. Firefly luciferin is found in fireflies and click beetles, and in addition to luciferase requires ATP as a cofactor to undergo oxidation. Coelenterazine is found in many marine organisms, including *Renilla*, *Vargula*, ctenophores, and shrimps, although unlike firefly luciferin, it does not require ATP for the luminescence reaction. Among these systems, NanoLuc (NLuc, also known as NanoKAZ [7]), a luciferase utilizing coelenterazine originating from *Oplophorus*, is one of the most widely used luciferases for in vivo bioimaging due to its small size, brightness, and ATP independence [8].

Given that these luciferases can emit light without excitation induced by an external light source, the use of a luminescence system should be effective in plant, for which fluorescence detection is typically difficult owing to the autofluorescence of chlorophyll. However, owing to reduced transmission through thick tissues, signals are often undetectable because of diminished brightness. Indeed, Ow *et al*. reported that it was necessary to perform a 24 h exposure in order to detect the luminescence of firefly luciferase expressed in tobacco cells [9]. Therefore, there is a strong demand for a more useful luminescence system that produces brighter signals for monitoring various biological events in plants.

To overcome the aforementioned barriers, it is necessary to take into account differences in the biological optical window among organisms for in vivo imaging. In addition to the shorter wavelength of light absorbed by vitamins and infrared light absorbed by water molecules, cells and tissues contain numerous types of molecules that can absorb visible light. In animals, light wavelengths ranging between 650 and 900 nm are considered to constitute a preferable optical window, as hemoglobin absorbs light ranging from 400 to 650 nm. In terrestrial plants, phytochrome and chlorophyll *a* and *b* block visible light which is absorbed for reactions related to development and photosynthesis [10,11]. On the basis of these considerations, it is apparent that the optimal luciferase detection system would differ depending on the organisms evaluated.

For this purpose, several approaches have been reported that facilitate adjustment of the emission wavelength of luminescence systems based on a modification of substrates and enzymes. With regards to substrates, a shift in the wavelength has been achieved by modifying the chemical structure of the substrate [12,13], while still enabling recognition of the modified structure by the wild-type enzyme. For luciferase enzymes, a basic approach involves the identification of novel luciferase genes in organisms emitting different colored luminescence [14]. Other approaches, include generating point mutations in wild-type luciferase [15] and shifting the wavelength by connecting fluorescent proteins using the bioluminescence resonance energy transfer (BRET) approach. One such example are the Nano-lanterns that have been shown to harness BRET to utilize fluorescent proteins that emit the color of interest [16–18]. For example, Suzuki *et al*. reported on the generation of bright Nano-lantern variants of differing color, referred to as enhanced Nano-lanterns, which are chimeras of NLuc and fluorescent proteins of different color [19]. These enhanced Nano-lanterns are sufficiently bright to be detectable as single molecules and can also be tracked as fluorescent proteins.

In this study, we sought to determine the optimal luciferase reporter system in plant tissues. We initially characterized the luminescence intensities of various types of luciferase reporter system in cultured *Arabidopsis thaliana* cells, and on the basis of our analyses, we identified green enhanced nano-lantern (GeNL)–furimazine as the brightest luciferase reporter in *A*. *thaliana* cells. The spectral peak of GeNL–furimazine luminescence was found to be located in the range of optical transparency for *A*. *thaliana* cells. Moreover, *A*. *thaliana* plants expressing GeNL with furimazine emitted strong luminescence, particularly in the root tissue. We thus speculate that GeNL–furimazine would be the most suitable luciferase reporter system for use in the analysis of a range of different terrestrial plants.

## Methods

### Plasmid construction

We used pCAMBIA-N-GUS as the backbone vector to express luciferase genes under the control of a 35S promoter. To construct pCAMBIA-N-GUS, the NOS promoter was amplified from pRI201 and cloned into the pCAMBIA 1305.2 vector (Marker Gene Technologies Inc., Eugene, OR, USA) partially digested with HindIII and XhoI, using an In-Fusion HD Cloning Kit (TaKaRa Bio., Shiga, Japan). The individual luciferase gene was PCR amplified and cloned into pCAMBIA-N-GUS at NcoI/SpeI and PmlI sites by In-Fusion cloning. The cDNAs of nnH3H and nnLuz were codon optimized for *A*. *thaliana* and synthesized (FASMAC, Kanagawa, Japan). The amino acid sequences of nnH3H, nnLuz, and GeNL are the same as those described in previous studies [19–22], although they differ with respect to the nucleic acid sequences. Details of the sequences are presented in S1 and S2 Notes.

### Cell culture and *Agrobacterium*-mediated transformation of T87 cells

The *A*. *thaliana* T87 strain (RIKEN Bio Resource Center, Ibaraki, Japan) was maintained and transformed with *Agrobacterium* (*Rhizobium radiobacter*) according to a previously described protocol [23]. Briefly, the T87 cells were co-cultured with *Agrobacterium* cells carrying the binary plasmids for 2 d and selected by plating on hygromycin-containing agar. Hygromycin-resistant calli were pooled and then transferred into liquid NT1 culture medium (30 g/L sucrose, 0.1 mM KH_2_PO_4_, 1× Murashige–Skoog Salt Mixture and Vitamins, 2 μM 2,4-dichlorophenoxyacetic acid, pH 5.8 adjusted with KOH) containing hygromycin.

### *Agrobacterium*-mediated transformation of *Arabidopsis* plants

*A*. *thaliana* (Col-0 strain; Inplanta Innovations, Kanagawa, Japan) plants were cultivated at 23°C under constant light. A flower bud was dipped in a culture of *Agrobacterium* cells carrying the binary reporter plasmid cell suspension containing 5% w/v sucrose, and 0.025% Silwet L-77 (Biomedical Science, Tokyo, Japan) for 3 min. After inoculation, the plant was placed under a cover to maintain high humidity and cultivated overnight under relatively weak light conditions. Subsequently, the plant was cultivated under normal conditions until seed formation. Seeds were collected and sown on a B5 agar plate (0.5% agar, 20 g/L glucose, 0.6 g/L MES, 1× Gamborg’s B5 Salt Mixture, 1× Murashige–Skoog Vitamins, pH 5.7 adjusted with KOH) supplemented with 200 μg/mL cefotaxime and 20 μg/mL hygromycin. After germination of the rhizome, plants (T_1_) emitting green fluorescence were transferred onto soil (vermiculite). T_2_ generation plants were used in our experiments.

### Analysis of T87 cell luminescence

We used the same numbers of cells for each reporter type in all luminescence experiments. Fifty microliters of reporter T87 cell culture of 1 μL packed cell volume (PCV) suspended in NT1 medium was transferred to a white 96-well plate (Greiner Bio-One, Kremsmünster, Austria). To detect NLuc activity, 5 μL of Nano-Glo luciferase assay reagent (Promega, Madison, WI, USA; containing furimazine) for NLuc, secNLuc, GeNL, and secGeNL was added to the cell culture and mixed. Concomitantly, to detect firefly luciferase activity, 5 μL of Dual-Glo luciferase reagent (Promega; containing d-luciferin) for FLuc and secFLuc was added to the cell culture and mixed. Luminescence was immediately measured using the ChemiDoc XRS+ system (BioRad, Hercules, CA, USA). To examine the effect of pH on GeNL luminescence activity, the pH of NT1 medium was adjusted accordingly using sodium phosphate buffer (final concentration 20 mM). To quantify the transmittance rate of luminescence, the white 96-well plate seeded with the reporter T87 cells was covered with a transparent 96-well plate containing either 100 μL of wild-type T87 cell culture of 50 μL PCV suspended in NT1 medium or 100 μL of NT1 medium. Transmittance rate was calculated as the transmitted luminescence intensity in the presence of the layer of wild-type T87 cells, divided by that in the absence of the layer. To image the transmitted luminescence, the white 96-well plate containing the reporter T87 cells was covered with a 35 mm dish containing either 2500 μL of wild-type T87 cell culture of 1250 μL PCV suspended in NT1 medium or 2500 μL of NT1 medium (please also refer to S1 Fig). To record a video, 50 μL of reporter T87 cell culture of 10 μL packed cell volume (PCV) suspended in NT1 medium was transferred to a white 96-well plate, to which 20 μL of Nano-Glo live-cell assay reagent (Promega) was added, followed by mixing. A color movie was acquired using a Galaxy S8 cell phone camera (Samsung, Suwon, Korea) without further image processing. To compare the luminescence intensity among nnH3H-nnLuz, FLuc, and GeNL, 5 μL of 1 mM hispidin (Fujifilm Wako, Tokyo, Japan), 5 μL of Dual-Glo luciferase reagent (Promega), or 5 μL of Nano-Glo luciferase assay reagent (Promega) was added to each cell culture and mixed. Images were obtained at 1, 10, and 30 min after substrate addition.

### Measurement of luminescence spectra

A 3 mL volume of reporter T87 cell culture of 150 μL PCV suspended in NT1 medium containing 2% v/v FP003 solution (FCeM Advance Preparation Kit500; Nissan Chemical Industries, Tokyo, Japan) was transferred to a quartz cuvette. Three hundred μL of Nano-Glo luciferase assay reagent (Promega) or 60 μL of 5 mM coelenterazine-h (Fujifilm Wako) were added to the cell culture and mixed. Luminescence spectra were immediately measured using an FP-8300 spectrofluorometer (Jasco, Tokyo, Japan) at 25°C with the excitation light source turned off (emission bandwidth, 5 nm; scan speed, 1000 nm/min). Spectral data were scanned 20 times and integrated to reduce the noise. The spectra thus obtained were normalized to the peak height for each reporter cell type in the range of 400 to 700 nm. The BRET ratio is defined as the ratio of acceptor (GeNL: 520 nm) and donor (NLuc: 450 nm) luminescence intensities.

### Measurement of absorption spectra

One hundred microliter volumes of wild-type T87 cell culture of 50 μL PCV suspended in NT1 medium were transferred to the wells of a transparent 96-well plate (AS ONE, Osaka, Japan). Absorption spectra were recorded using a microplate reader (Infinite 200 Pro; Tecan, Zurich, Switzerland). Spectral data were recorded 10 times and integrated to reduce the noise.

### Analysis of the fluorescence and luminescence of *Arabidopsis thaliana* reporter plants

For fluorescence analysis, *A*. *thaliana* reporter plants were transferred to a B5 agar plate. Images were obtained using the ChemiDoc XRS+ system (BioRad) using a GelGreen filter (560/50 nm). For luminescence analysis, *A*. *thaliana* reporter plants were transferred to a transparent 48-well plate (AS ONE) containing 300 μL of NT1 medium and 10 μL of Nano-Glo live cell assay reagent (Promega) with or without detergent. Luminescence was quantified using the ChemiDoc XRS+ system (BioRad).

### Quantification and statistical analyses

All data were processed and analyzed using Excel 2019 (Microsoft, Redmond, WA, USA), and all measurements are presented as the mean ± standard error (SE) of more than three tests. Sample sizes are indicated in the figure legends. Statistical significance was evaluated using an independent-samples Student’s *t* test, and statistically significant values were defined as ***p < 0.001.

## Results

### Luminescence in cultured *Arabidopsis thaliana* cells

**Fig 1:**
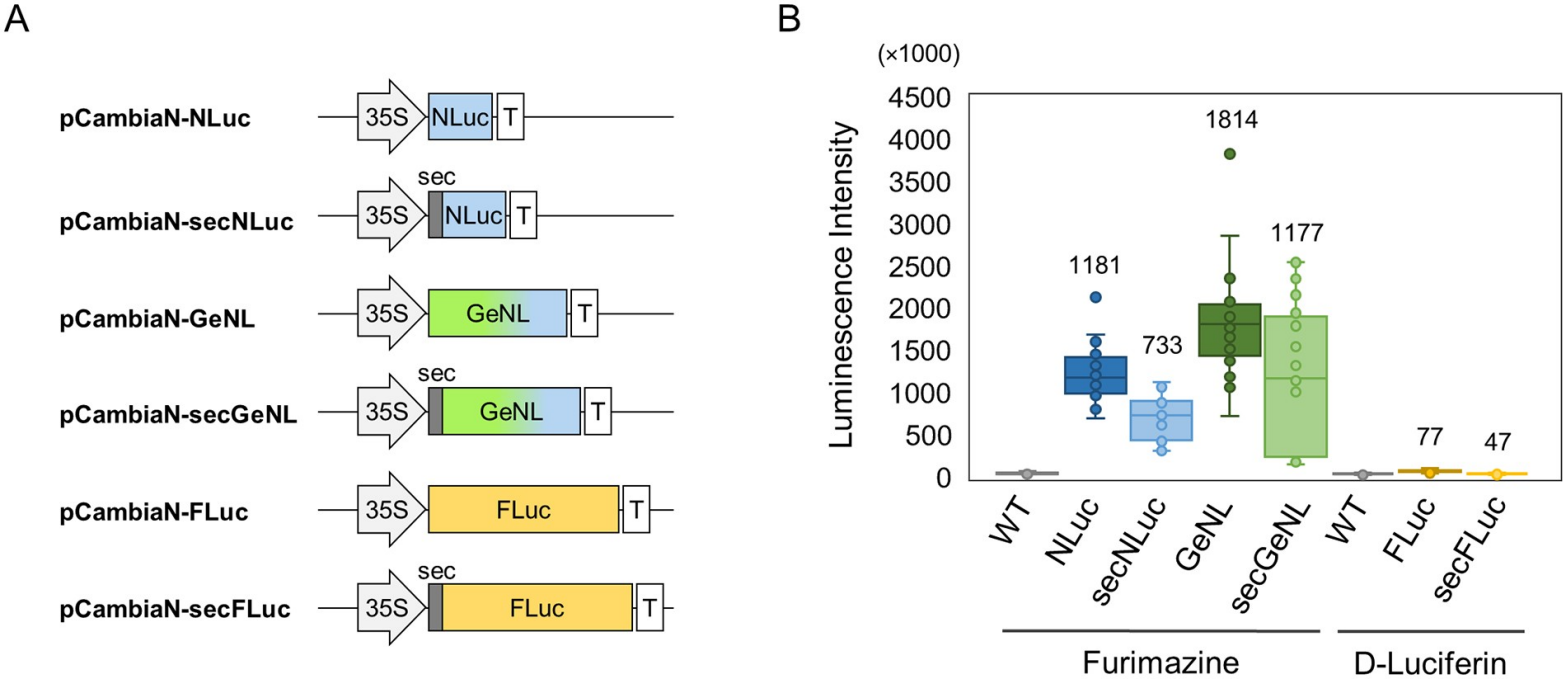
Design of luciferase reporter T87 cells. (A) A schematic diagram showing the vector constructs used in this study. 35S, sec, and T indicate the 35S promoter, secretory signal, and terminator polyadenylation signal, respectively. (B) Box plot of luminescence intensities for luciferase reporter *Arabidopsis thaliana* T87 cells on addition of furimazine or d-luciferin. Nano-Glo luciferase assay system and Dual-Glo luciferase assay system were used for NLuc and FLuc, respectively. The data are from three individual experiments with biological octuplicates; n = 24. Values indicate averages.

For luminescent imaging in *A*. *thaliana* cells, we examined *Photinus pyralis* firefly luciferase (FLuc) and NLuc, which is among the brightest luciferases. To detect NLuc activity, we used furimazine, which has been identified as an optimal substrate for NLuc in terms of high stability and low autoluminescence [8]. We also prepared a green enhanced Nano-lantern (GeNL), the emission of which is red-shifted, by fusing the mNeonGreen protein as a BRET acceptor to NLuc [19]. Given the presence of plant cell walls, which restrict the entry of exogenous substances, cellular uptake efficiency is assumed to be low compared with animal cells. Therefore, we also prepared a construct in which an extracellular secretory signal was added to each luciferase (secFLuc, secNLuc, and secGeNL in Fig 1A). Secreted luciferase would enable the detection of luminescence in living cells independently of substrate permeability across the cell membrane. To express these luciferase genes in *A*. *thaliana* cells, we constructed a binary vector for *Agrobacterium* (Fig 1A). *Agrobacterium* cells carrying these vectors were then used to infect T87 cells, a representative *A*. *thaliana* cell line, to obtain luciferase reporter cell lines.

**Fig 2:**
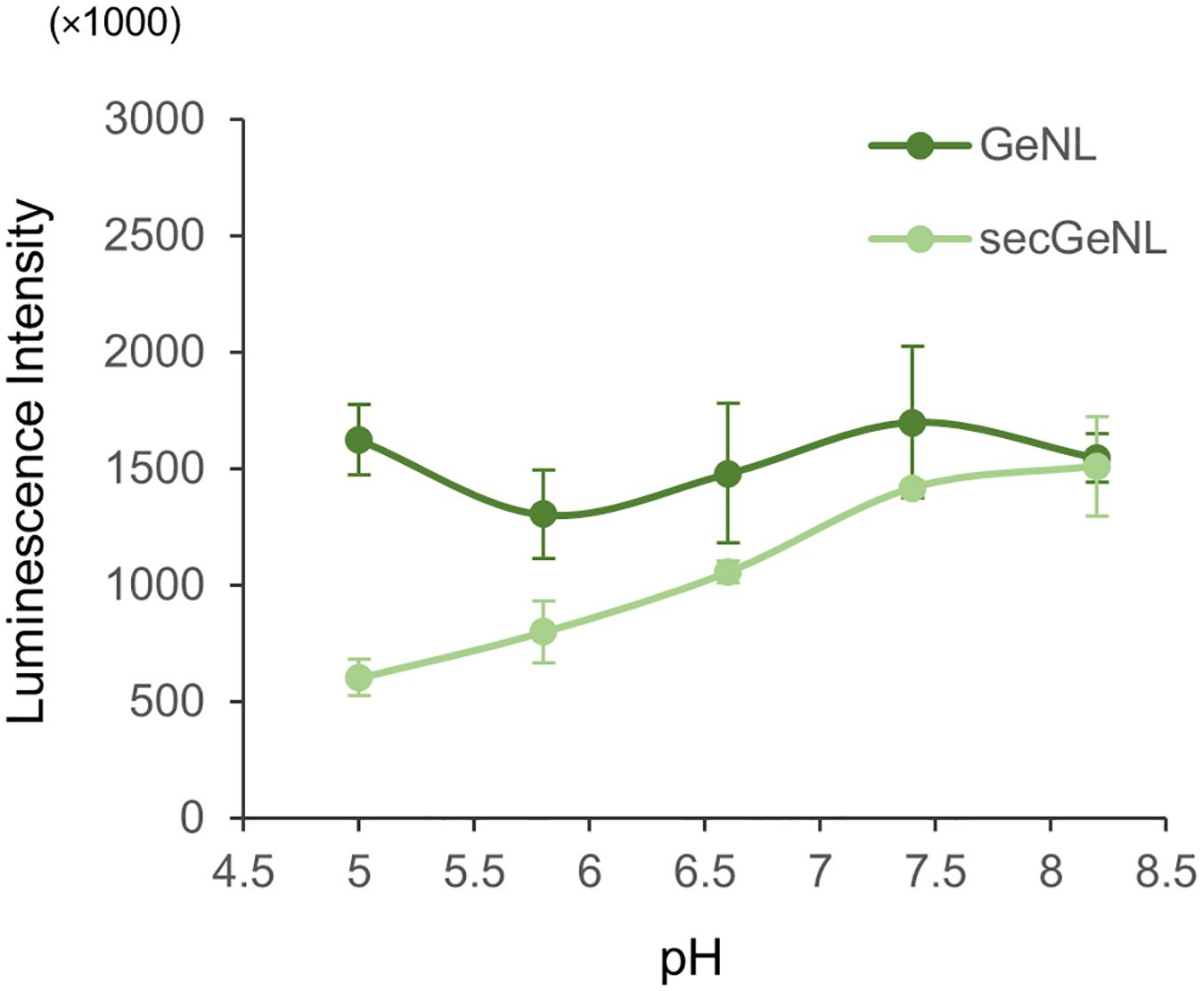
Effect of pH conditions on GeNL reporter activity. The luminescence intensities of GeNL and secGeNL reporter cells were determined on addition of furimazine under different pH conditions (5.0, 5.8, 6.6, 7.4, and 8.2). Values shown are the mean ± SE; n = 3.

Luciferase assays were performed using these reporter cell lines. Given that the optimal substrate and its optimal concentration differ depending on each luciferase used, different optimal conditions were used for each luciferase evaluated. To assess luciferase-generated luminescence independent of wavelength, we detected luminescence without applying optical filters. As shown in Fig 1B, GeNL and NLuc showed higher luminescence than FLuc. A comparison of GeNL and NLuc revealed that the luminescence of GeNL was slightly higher. Although the relationship between these luciferases was not altered when a secretory signal was used, the secreted luciferases were characterized by reduced signal intensities. This difference in signal intensity could be attributable to differences in the pH between the intra- and extracellular environments. The extracellular environment of the plant cells in the present study was acidic, given that the pH of the culture medium for T87 cells is 5.8, whereas the intracellular pH is approximately 7.4. Accordingly, since the optimal pH for NLuc is approximately 8.0, the luminescence intensity of the secreted luciferase could be increased by adjusting the pH of the medium, and thus we examined the effect of pH on luminescence intensity using the GeNL and secGeNL reporter cells. As shown in Fig 2, the luminescence intensity of GeNL cells was essentially unaffected by changes in pH, whereas secGeNL cells showed an increase in luminescence intensity with increasing pH, and at pH 8.2 exhibited the same intensity as GeNL cells. These results indicated that GeNL is a suitable luciferase that can be used in luciferase reporter systems in *A*. *thaliana* cells and that by applying weak basic pH conditions, the luciferase activity of secreted GeNL can be further enhanced.

### Luminescence spectra in cultured *Arabidopsis thaliana* cells

**Fig 3:**
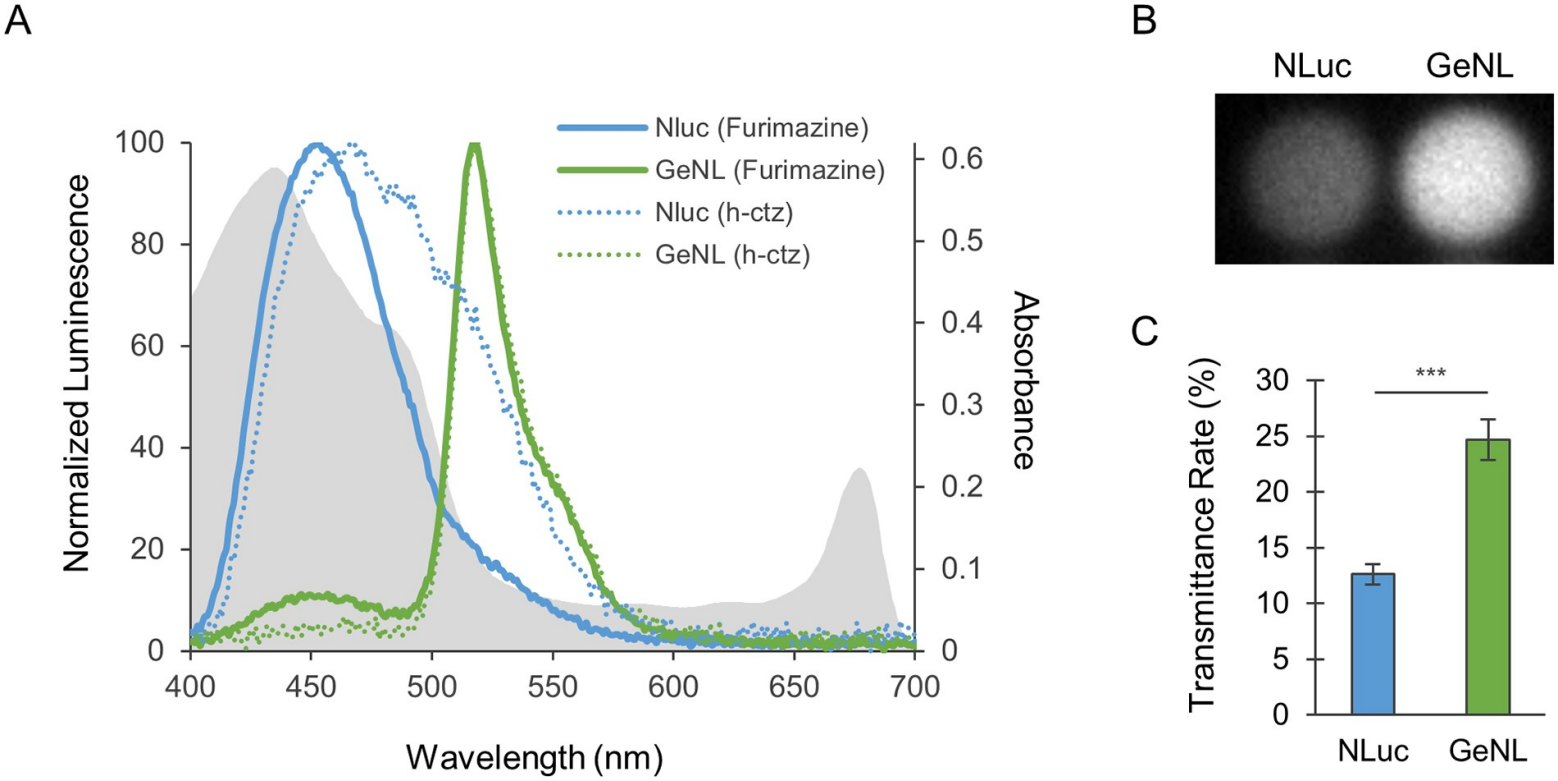
Detection of reporter cell luminescence through a layer of T87 cells. (A) Normalized emission spectra of NLuc (blue) and GeNL (green) reporter *Arabidopsis thaliana* T87 cells on addition of furimazine. The emission spectra of NLuc (blue dotted line) and GeNL (green dotted line) cells on the addition of coelenterazine h are also shown. The gray shade indicates the absorbance spectrum of wild-type T87 cells. The spectra were normalized to peak height for each reporter cell type in the range 400 to 700 nm. (B) Luminescence images of NLuc (blue) and GeNL (green) reporter T87 cells on addition of NLuc luciferin through a layer of wild-type T87 cells. (C) Transmittance rates of reporter cells covered with a layer of T87 cells. Transmittance rates were calculated as the transmitted luminescence intensity in the presence of a layer of wild-type T87 cells, divided by that in the absence of a layer. The values shown are the mean ± SE; n = 4. Student’s *t* test was performed. ***p < 0.001.

There are various molecules that interfere with the detection of luminescence in cells. Terrestrial plants are known to be rich in chlorophyll *a* and *b*, which absorb light in the 400–500 nm and 600–700 nm ranges [11]. As shown in Fig 3A (gray shade), T87 cells have a large and small absorption peak at 400–500 nm and approximately 670 nm, respectively. This finding is consistent with the reported absorption spectra of chlorophyll *a* and *b*. Subsequently, we measured of the luminescence spectra of both NLuc and GeNL cells, which showed high luminescence intensities (Fig 1). As shown in Fig 3A, NLuc (blue solid line) and GeNL (green solid line) showed emission peaks at approximately 450 nm and 520 nm, respectively. Merged spectra results revealed that in contrast to GeNL, NLuc luminescence can be readily absorbed by *A*. *thaliana* cells. We also evaluated the energy transfer efficiency of GeNL in T87 cells by calculating the BRET ratio. Suzuki *et al*. calculated the BRET ratio of GeNL expressed in *Escherichia coli* as the ratio of peak intensity at 520/450 nm and reported it to be approximately 5.5 [19]. Using the same calculation, we obtained a BRET ratio of 8.7 for T87 cells, thereby indicating that the BRET of GeNL occurs normally even in a plant cell background.

Given that the emission peak of NLuc with coelenterazine is known to be red-shifted from that with furimazine [13], we also measured the luminescence spectra of those cells with coelenterazine h, a bright coelenterazine derivative, as a substrate. As shown in Fig 3A, the emission peak of NLuc cells with coelenterazine h (468 nm) was red-shifted from that observed with furimazine (453 nm), whereas the emission peak of GeNL cells was virtually unchanged as a consequence of changing substrate (517 nm to 519 nm). These results accordingly indicated the possibility that a wavelength shift could also be achieved by changing the substrate, although the wavelength shift of NLuc with coelenterazine h was not sufficient for it to fall within the optical window of T87 cells.

To evaluate whether GeNL light can penetrate through T87 cells, we measured the optical transmittance rates of NLuc and GeNL luminescence through T87 cells using furimazine. Luminescence measurements were performed using the reporter cells in a 96-well plate covered with a layer of wild-type T87 cells or medium only (S1 Fig). Transmittance rate was defined as the transmitted luminescence intensity in the presence of the layer of wild-type T87 cells, divided by that in the absence of the layer. As shown in Fig 3B and 3C, only approximately 12% of the luminescence of the NLuc cells was transmitted, whereas approximately 25% of that of GeNL cells was transmitted. These results indicate that GeNL–furimazine luminescence is significantly more transmittable through terrestrial plant cells than is the luminescence of NLuc–furimazine. Moreover, as shown in S1 Movie, the luminescence of GeNL cells was sufficiently strong to be imaged even with a cell phone. Thus, GeNL–furimazine appears to be the most effective system in reporter assays in planta, wherein light needs to pass through neighboring cells.

### Detection of GeNL luminescence in planta

**Fig 4:**
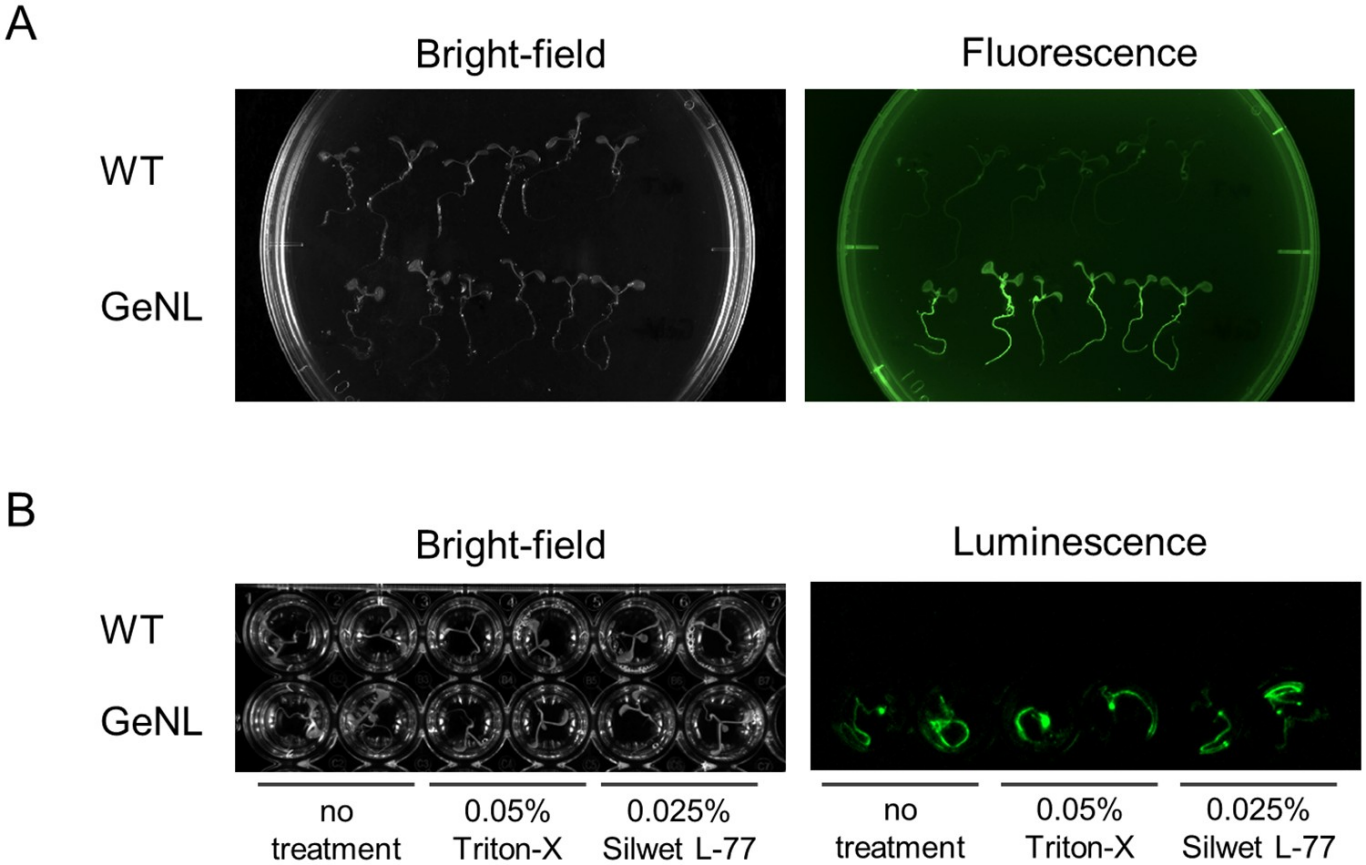
Luminescence imaging of GeNL expressing *Arabidopsis thaliana*. (A) Fluorescence image of GeNL-expressing *Arabidopsis thaliana* in a 10 cm dish. The image was obtained using a GelGreen filter (560/50 nm). (B) Luminescence image of GeNL-expressing *A*. *thaliana* in a 48-well plate on addition of furimazine. WT indicates wild-type *A*. *thaliana* plants.

On the basis of our observation that the GeNL–furimazine combination showed the best luminescence properties in cultured cell experiments, we subsequently evaluated GeNL imaging with furimazine in planta. Using the *Agrobacterium*-mediated transformation method, we produced *A*. *thaliana* plants expressing GeNL. As these plants emit mNeonGreen-derived yellow-green fluorescence under blue light excitation, transformants expressing the reporter gene could be visibly selected (Fig 4A). When furimazine was added to these transformants, strong luminescence was observed in the entire plant (Fig 4B), and the intensity was sufficiently high to enable detection with the naked eye. Furthermore, we found the detectable luminescence to be high in roots but lower in stems and leaves, thereby indicating that for the analysis of terrestrial parts, substrate uptake in these tissues must be enhanced. These results, thus, indicated that GeNL–furimazine would be a useful luminescent reporter in planta.

## Discussion

In the present study, we sought to determine the optimal luminescent reporter for plants using cultured *A*. *thaliana* cells and whole plants. Terrestrial plants, including *A*. *thaliana*, contain large amounts of chlorophyll *a* and *b* that absorb light at blue (400–500 nm) and red (600–700 nm) wavelengths. Given that the luminescence peak of NLuc–furimazine occurs at 453 nm and overlaps with the absorption spectrum of chlorophyll (400–500 nm), it showed low transmittance in plant cells and is thus difficult to detect. Therefore, we focused on further evaluating the luminescence of GeNL with a peak emission at 517 nm that would differ sufficiently from the absorption wavelength band of chlorophyll *a* and *b* by BRET. As expected, the luminescence of GeNL showed greater transmission than that of NLuc. Moreover, bright GeNL luminescence was detected in both cultured cells and plants. Considering that a majority of terrestrial plants contain chlorophyll *a* and *b*, GeNL could be a powerful luminescence tool in plant biology research. Nevertheless, the fact that pigment composition varies among photosynthetic organisms needs to be taken into consideration. For example, brown algae contain chlorophyll *a* and *c* and fucoxanthin, whereas red algae contain chlorophyll *a*, phycoerythrin, and phycocyanin [24,25]. Fucoxanthin, phycoerythrin, and phycoerythrin absorb blue-green light (370–520 nm), green-yellow light (480–580 nm), and red light (550–650 nm), respectively, and thus luciferases with different emission wavelengths would be necessary when studying these organisms. In addition to GeNL, Suzuki *et al*. succeeded in producing luciferases with various emission wavelengths from blue to red, by fusing various fluorescent proteins to NLuc. For example, OeNL (orange enhanced Nano-lantern) with an emission peak at 565 nm might be more suitable for use in brown algae, whereas CeNL (cyan enhanced Nano-lantern) with an emission peak at 475 nm might be preferable for examining red algae. Therefore, the BRET strategy employed in the present study could also prove to be effective for application in other organisms.

In plant biology, the GUS reporter is one of the most extensively employed reporter systems. The GUS gene encodes β-glucuronidase that catalyzes the hydrolysis of β-d-glucuronic acid residues from the non-reducing end of mucopolysaccharides. GUS-expressing cells and tissue can be visualized either by staining with a blue dye using X-Gluc or by the emitted fluorescence via utilization of the 6-chloro-4-methylumbelliferyl-β-d-glucuronide (CMUG) fluorogenic substrate [23,26]. Although these techniques enable the spatial visualization and quantification of gene expression, they are both invasive and time-consuming. Furthermore, these methods are often associated with high background signals from endogenous GUS activity or the autofluorescence of chlorophyll *a* and *b*. Given that there is no need to fix samples and the furimazine substrate is non-toxic, the GeNL reporter system constructed in the present study can be used to visualize and simultaneously quantify gene expression non-invasively. In addition, the measurement of luminescence takes only a few minutes and is highly quantitative. Furthermore, as there is no luciferase homolog in terrestrial plants, the background signal is low. Recently, Urquiza-García *et al*. reported on a luminescence reporter system using NLuc [27]; however, as demonstrated in the present study, most of the emission wavelength of NLuc tends to be absorbed by plant cell components (Fig 3). By fusing a BRET acceptor exhibiting a longer emission wavelength, the luminescence of GeNL can be twice as transmissive through plant cells as NLuc. More recently, Mitiouchkina *et al*. and Khakhar *et al*. have generated plants with self-sustained luminescence by incorporating a recently discovered gene for a fungal luciferase and the genes for total biosynthesis of the corresponding substrate into plants such as tobacco and *Arabidopsis* [20–22]. However, although the fungal luciferase system emits a greenish luminescence that falls within the plant optical window, the light intensity of the fungal luciferase is not as high as that of GeNL (S2 Fig). Collectively, our observations indicate that the GeNL reporter system could make a valuable contribution to the biological analysis of cellular functions and prove useful in a range of plant applications.

## Conclusion

In conclusion, in this study, using cultured *A*. *thaliana* cells and whole plants, we sought to determine the most suitable luminescence system for use in reporter assays in plants. We accordingly found that the NLuc–furimazine enzyme–substrate combination was characterized by very bright luminescence in plant cells, although most of this luminescence was absorbed by plant cellular components, including chlorophyll *a* and *b*. Comparatively, using the GeNL–furimazine system, which is characterized by a longer emission wavelength, we succeeded in constructing a reporter system that is brighter and more transmissive compared with that of NLuc–furimazine. Moreover, *A*. *thaliana* plants expressing GeNL emitted strong luminescence with furimazine, which could be detected with the naked eye. These results indicated that GeNL–furimazine could function as an exceptionally useful reporter for visualizing gene expression in plants. This simple and effective reporter system should contribute to the biological analysis of genes and plant functions both in vitro and in planta.

## Supporting information

S1 Fig Schematic illustration of the luminescence transmission assay. Wild-type *Arabidopsis thaliana* T87 cells were seeded in a 35 mm dish and luciferase reporter cells in a 96-well plate covered with a 35 mm dish containing either the wild-type T87 cells or medium only. Transmitted luminescence was detected using a detector positioned above the dish.
(TIF)

S2 Fig Comparison of luminescence intensities among three bioluminescence systems. (A) Schematic diagram showing the pCambiaN-nnH3H-nnLuz vector construct. 35S, 2A, and T indicate the 35S promoter, 2A self-cleaving peptide of porcine teschovirus-1, and terminator polyadenylation signal, respectively. (B) Luminescence images and intensities of luciferase reporter T87 cells on addition of hispidin, d-luciferin, or furimazine. nnH3H-nnLuz cells (left well), FLuc cells (center well) and GeNL cells (right well) were used as reporter cells. Values below indicate the luminescence intensities of each well (×10^3^).
(TIF)

S1 Movie Luminescence movie using *Arabidopsis thaliana* T87 culture cells. Furimazine was added to the cell culture and mixed. Left: empty well, Center: T87 expressing GeNL, Right: wild-type T87 cells. Green luminescence was observed using the naked eye.
(MP4)

S1 Note DNA sequence of the pCambiaN-GeNL plasmid. (PDF)

S2 Note Amino acid sequences of individual luciferases. (PDF)
