# Supplementary figures and images for: Bioluminescent imaging of Arabidopsis thaliana using an enhanced Nano-lantern luminescence reporter system
Source: PLoS One. 2020 Jan 3;15(1):e0227477. doi: 10.1371/journal.pone.0227477 (PMC6941820; doi:10.1371/journal.pone.0227477)

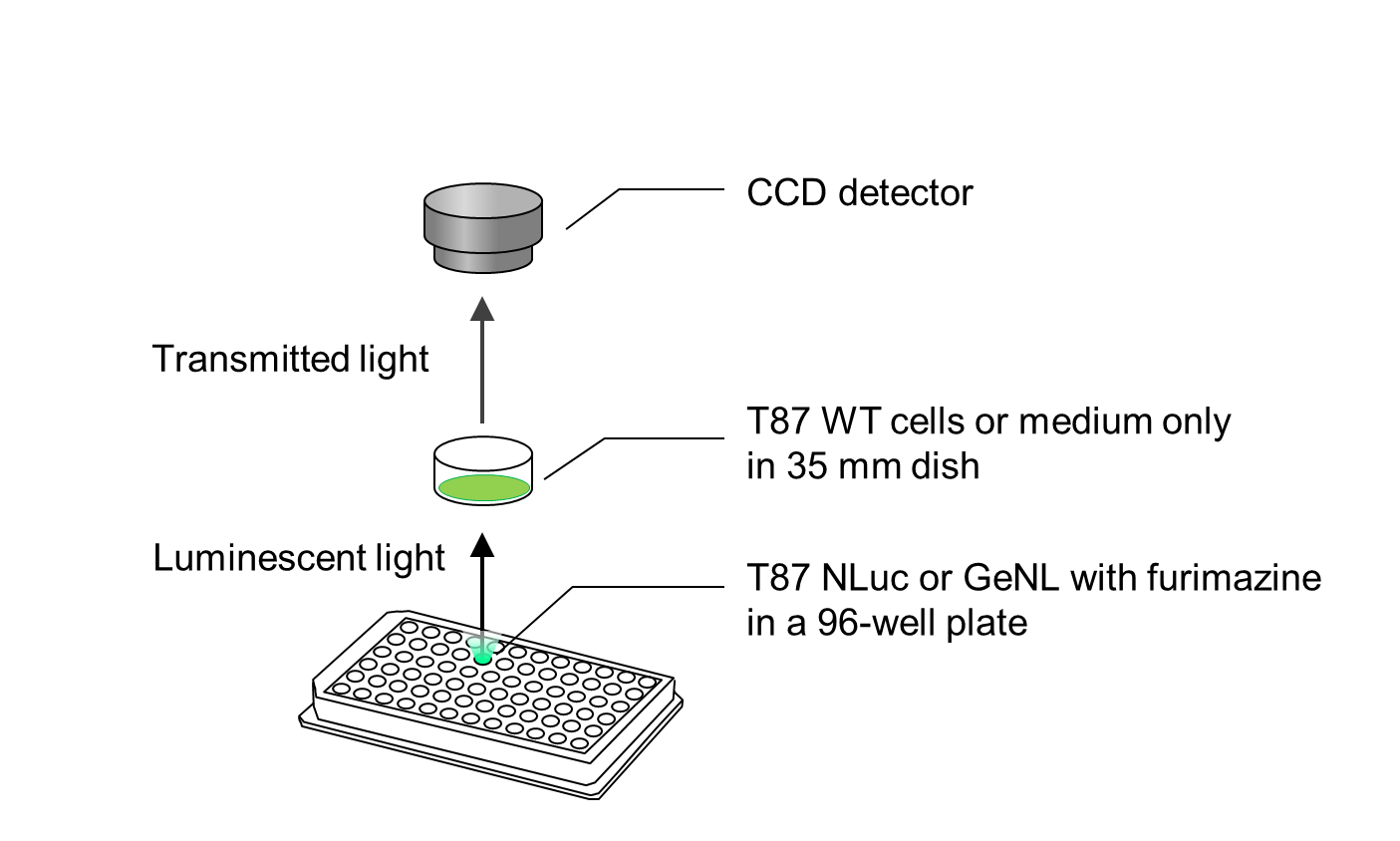

Supplement: S1 Fig — Wild-type Arabidopsis thaliana T87 cells were seeded in a 35 mm dish and luciferase reporter cells in a 96-well plate covered with a 35 mm dish containing either the wild-type T87 cells or medium only. Transmitted luminescence was detected using a detector positioned above the dish. (TIF) [file pone.0227477.s001.TIF]

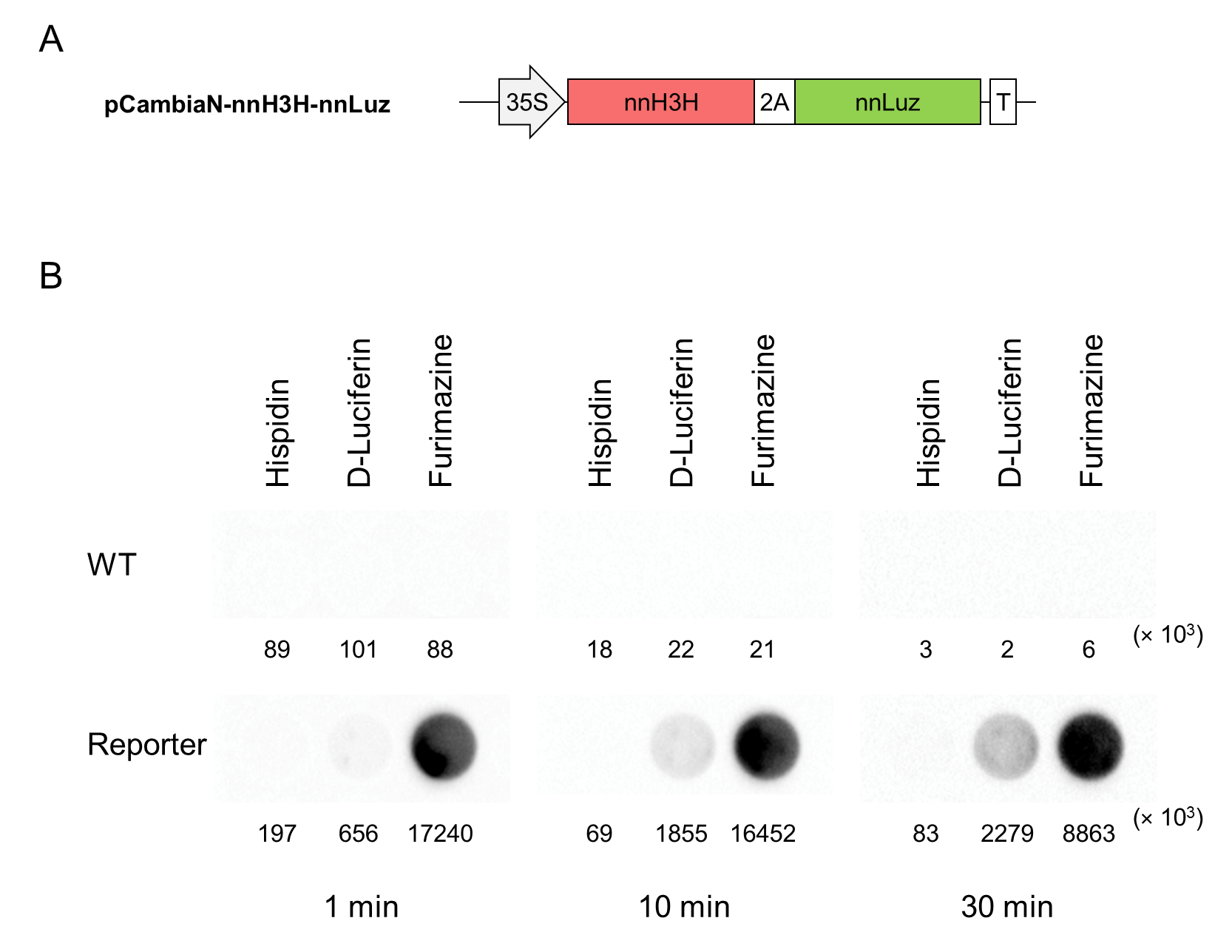

Supplement: S2 Fig — (A) Schematic diagram showing the pCambiaN-nnH3H-nnLuz vector construct. 35S, 2A, and T indicate the 35S promoter, 2A self-cleaving peptide of porcine teschovirus-1, and terminator polyadenylation signal, respectively. (B) Luminescence images and intensities of luciferase reporter T87 cells on addition of hispidin, d-luciferin, or furimazine. nnH3H-nnLuz cells (left well), FLuc cells (center well) and GeNL cells (right well) were used as reporter cells. Values below indicate the luminescence intensities of each well (×103). (TIF) [file pone.0227477.s002.TIF]
