# Supplementary material for: Bioluminescent imaging of Arabidopsis thaliana using an enhanced Nano-lantern luminescence reporter system
Source: PLoS One. 2020 Jan 3;15(1):e0227477. doi: 10.1371/journal.pone.0227477 (PMC6941820; doi:10.1371/journal.pone.0227477)
Supplement: S2 Note — (PDF) [file pone.0227477.s005.pdf]

S2 Note| Amino acid sequences of individual luciferases

FLuc: Firefly luciferase, originally from pGL4 (Promega)

MEDAKNIKKGPAPFYPLEDGTAGEQLHKAMKRYALVPGTIAFTDAHIEVDITYAEYFEMSVRLAEAMKRYGLNTNHRIVVCSENSLQFFMPVLGALFIGVAVAPANDIYNERELLNSMGISQPTVVFVSKKGLQKIL
NVQKKLPIIQKIIIMDSKTDYQGFQSMYTFVTSHLPPGFNEYDFVPESFDRDKTIALIMNSSGSTGLPKGVALPHRTACVRFSHARDPIFGNQIIPDTAILSVVPFHHGFGMFTTLGYLICGFRVVLMYRFEEELFL
RSLQDYKIQSALLVPTLFSFFAKSTLIDKYDLSNLHEIASGGAPLSKEVGEAVAKRFHLPGIRQGYGLTETTSAILITPEGDDKPGAVGKVVPFFEAKVVDLDTGKTLGVNQRGELCVRGPMIMSGYVNNPEATNAL
IDKDGWLHSGDIAYWDEDEHFFIVDRLKSLIKYKGYQVAPAELESILLQHPNIFDAGVAGLPDDDAGELPAAVVVLEHGKTMTEKEIVDYVASQVTTAKKLRGGVVFVDEVPKGLTGKLDARKIREILIKAKKGGKI
AV

secFLuc: Firefly luciferase with added secretory signal

MATTKHLALAILVLLSIGMTTSARTLLDLRNRRTSEDAKNIKKGPAPFYPLEDGTAGEQLHKAMKRYALVPGTIAFTDAHIEVDITYAEYFEMSVRLAEAMKRYGLNTNHRIVVCSENSLQFFMPVLGALFIGVAVA
PANDIYNERELLNSMGISQPTVVFVSKKGLQKILNVQKKLPIIQKIIIMDSKTDYQGFQSMYTFVTSHLPPGFNEYDFVPESFDRDKTIALIMNSSGSTGLPKGVALPHRTACVRFSHARDPIFGNQIIPDTAILSV
VPFHHGFGMFTTLGYLICGFRVVLMYRFEEELFLRSLQDYKIQSALLVPTLFSFFAKSTLIDKYDLSNLHEIASGGAPLSKEVGEAVAKRFHLPGIRQGYGLTETTSAILITPEGDDKPGAVGKVVPFFEAKVVDLD
TGKTLGVNQRGELCVRGPMIMSGYVNNPEATNALIDKDGWLHSGDIAYWDEDEHFFIVDRLKSLIKYKGYQVAPAELESILLQHPNIFDAGVAGLPDDDAGELPAAVVVLEHGKTMTEKEIVDYVASQVTTAKKLRG
GVVFVDEVPKGLTGKLDARKIREILIKAKKGGKIAV

NLuc: NanoLuc, originally from pNL (Promega)

MVFTLEDFVGDWRQTAGYNLDQVLEQGGVSSLFQNLGVSVTPIQRIVLSGENGLKIDIHVIIPYEGLSGDQMGQIEKIFKVVYPVDDHHFKVILHYGTLVIDGVTPNMIDYFGRPYEGIAVFDGKKITVTGTLWNGN
KIIDERLINPDGSLLFRVTINGVTGWRLCERILA

secNLuc: NanoLuc with added secretory signal

MATTKHLALAILVLLSIGMTTSARTLLDLRNRRTSVFTLEDFVGDWRQTAGYNLDQVLEQGGVSSLFQNLGVSVTPIQRIVLSGENGLKIDIHVIIPYEGLSGDQMGQIEKIFKVVYPVDDHHFKVILHYGTLVIDG
VTPNMIDYFGRPYEGIAVFDGKKITVTGTLWNGNKIIDERLINPDGSLLFRVTINGVTGWRLCERILA

GeNL: Green enhanced Nano-lantern (fusion of mNeonGreen, which has been chemically synthesized to alter codons, and NanoLuc with optimized linkers)

MVSKGEEDNMASLPATHELHIFGSINGVDFDMVGQGTGNPNDGYEELNLKSTKGDLQFSPWILVPHIGYGFHQYLPYPDGMSPFQAAMVDGSGYQVHRTMQFEDGASLTVNYRYTYEGSHIKGEAQVKGTGFPADGP
VMTNSLTAADWCRSKKTYPNDKTIISTFKWSYTTGNGKRYRSTARTTYTFAKPMAANYLKNQPMYVFRKTELKHSKTELNFKEWQKAFTGFEDFVGDWRQTAGYNLDQVLEQGGVSSLFQNLGVSVTPIQRIVLSGE
NGLKIDIHVIIPYEGLSGDQMGQIEKIFKVVYPVDDHHFKVILHYGTLVIDGVTPNMIDYFGRPYEGIAVFDGKKITVTGTLWNGNKIIDERLINPDGSLLFRVTINGVTGWRLCERILA

secGeNL: GeNL with added secretory signal

MATTKHLALAILVLLSIGMTTSARTLLDLRNRRTSVSKGEEDNMASLPATHELHIFGSINGVDFDMVGQGTGNPNDGYEELNLKSTKGDLQFSPWILVPHIGYGFHQYLPYPDGMSPFQAAMVDGSGYQVHRTMQFE
DGASLTVNYRYTYEGSHIKGEAQVKGTGFPADGPVMTNSLTAADWCRSKKTYPNDKTIISTFKWSYTTGNGKRYRSTARTTYTFAKPMAANYLKNQPMYVFRKTELKHSKTELNFKEWQKAFTGFEDFVGDWRQTAG
YNLDQVLEQGGVSSLFQNLGVSVTPIQRIVLSGENGLKIDIHVIIPYEGLSGDQMGQIEKIFKVVYPVDDHHFKVILHYGTLVIDGVTPNMIDYFGRPYEGIAVFDGKKITVTGTLWNGNKIIDERLINPDGSLLFR
VTINGVTGWRLCERILA

nnH3H-2A-nnLuz: Luciferin-precursor hydroxylase (H3H), 2A self-cleaving peptide of porcine teschovirus-1, and luciferase (Luz) of *Neonothopanus nambi*

MASFENSLSVLIVGAGLGGLAAAIALRRQGHVVKIYDSSSFKAELGAGLAVPPNTLRSLQQLGCNTENLNGVDNLCFTAMGYDGSVGMMNNMTDYREAYGTSWIMVHRVDLHNELMRVALDPGGLGPPATLHLNHRV
TFCDVDACTVTFTNGTTQSADLIVGADGIRSTIRRFVLEEDVTVPASGIVGFRWLVQADALDPYPELDWIVKKPPLGARLISTPQNPQSGVGLADRRTIIIYACRGGTMVNVLAVHDDERDQNTADWSVPASKDDLF
RVFHDYHPRFRRLLELAQDINLWQMRVVPVLKKWVNKRVCLLGDAAHASLPTLGQGFGMGLEDAVALGTLLPKGTTASQIETRLAVYEQLRKDRAEFVAAESYEEQYVPEMRGLYLRSKELRDRVMGYDIKVESEKV
LETLLRSSNSAGATNFSLLKQAGDVEENPGPMRINISLSSLFERLSKLSSRSIAITCGVVLASAIAFPIIRRDYQTFLEVGPSYAPQNFRGYIIVCVLSLFRQEQKGLAIYDRLPEKRRWLADLPFREGTRPSITSH
IIQRQRTQLVDQEFATRELIDKVIPRVQARHTDKTFLSTSKFEFHAKAIFLLPSIPINDPLNIPSHDTVRRTKREIAHMHDYHDCTLHLALAAQDGKEVLKKGWGQRHPLAGPGVPGPPTEWTFLYAPRNEEEARVV
EMIVEASIGYMTNDPAGKIVENAK
